# Elevated red blood cell folate levels are associated with metabolic dysfunction-associated steatotic liver disease: results from NHANES 2017–2020

**DOI:** 10.3389/fphys.2025.1494863

**Published:** 2025-03-20

**Authors:** Xin Liao, Song Yu, Lin Wang, Ruyue Zhang, Ke Yu

**Affiliations:** ^1^ Department of General Medicine, The General Hospital of Western Theater Command, Chengdu, Sichuan, China; ^2^ Department of General Medicine, the Third Affiliated Hospital of Chengdu Medical College, Chengdu Pidu District People’s Hospital, Chengdu, Sichuan, China

**Keywords:** metabolic dysfunction-associated steatotic liver disease, red blood cell folate, folate, controlled attenuation parameters, National Health and Nutrition Examination Survey

## Abstract

**Introduction:**

Metabolic dysfunction-associated steatotic liver disease (MASLD) is the most prevalent chronic liver disease worldwide. However, the role of folate in MASLD remains controversial. This study aimed to investigate the association between two folate indicators [serum folate and red blood cell (RBC) folate] and MASLD prevalence using data from the 2017–2020 National Health and Nutrition Examination Survey (NHANES).

**Methods:**

A total of 3,879 participants without liver disease or significant alcohol consumption were included in the final analysis. Hepatic steatosis was assessed via transient elastography, with MASLD defined as a controlled attenuation parameter (CAP) ≥285 dB/m and the presence of at least one cardiometabolic risk factor. Logistic regression and generalized additive models (GAMs) were used to evaluate associations between folate levels and MASLD, with subgroup analyses stratified by age, gender, and body mass index (BMI).

**Results:**

After full adjustment for confounders, RBC folate exhibited a significant positive association with MASLD (OR = 1.111 and 95% CI: 1.015–1.216 per 1-unit increase). In contrast, serum folate showed a transient negative association in minimally adjusted models (OR = 0.869 and 95% CI: 0.802–0.941), which disappeared after further adjustments. Subgroup analyses confirmed that age, gender, and BMI did not modify the RBC folate–MASLD relationship.

**Discussion:**

These findings suggest that elevated RBC folate levels are independently associated with MASLD prevalence, whereas serum folate may lack clinical relevance due to susceptibility to confounding factors. RBC folate, as a stable biomarker of long-term folate status, may serve as a superior indicator for investigating folate–MASLD associations.

## 1 Introduction

Metabolic dysfunction-associated steatotic liver disease (MASLD) is one of the leading causes of chronic liver disease, affecting approximately 25% of the U.S. population and 30% of the global population (Zhang and Brandman, 2025). It is also the second most common indication for liver transplantation (Kwong et al., 2024). Historically, MASLD was referred to as non-alcoholic fatty liver disease (NAFLD). However, the exclusionary diagnostic criteria associated with NAFLD have been deemed inappropriate, prompting a redefinition of the condition. In 2020, an international panel of experts from 22 countries proposed the term “metabolic dysfunction-associated fatty liver disease (MAFLD)” (Eslam et al., 2020). Subsequently, in 2023, three major international liver associations introduced the new nomenclature “metabolic dysfunction-associated steatotic liver disease (MASLD)” to replace both NAFLD and MAFLD (Lazarus et al., 2024). Unlike NAFLD, MASLD more accurately characterizes the disease by emphasizing the relationship between fatty liver and metabolic dysfunction rather than simply categorizing it based on alcohol consumption (Fan et al., 2024). According to the recent Delphi Consensus Statement, MASLD is defined as steatotic liver disease (SLD) accompanied by at least one cardiometabolic risk factor (e.g., obesity, elevated blood glucose, elevated blood pressure, elevated triglycerides, or reduced high-density lipoprotein levels) and the absence of other apparent causes of SLD. Currently, the primary treatment for MASLD focuses on lifestyle modifications, including weight loss, exercise, reduced caloric intake, and decreased consumption of saturated fats and sugary beverages. However, no specific medications have been proven effective for MASLD treatment (Rinella et al., 2023).

Folate, a water-soluble B9 vitamin, serves as a coenzyme substrate for one-carbon transfer reactions, which are crucial for nucleic acid biosynthesis, methylation reactions, and sulfur-containing amino acid metabolism (Younossi, 2019). Given that MASLD is a relatively new concept, there are limited reports on the relationship between folate and MASLD. However, due to the overlap between MASLD and NAFLD, findings related to NAFLD can provide some insights. Nevertheless, clinical research on the association between folate and NAFLD has yielded contradictory results, and we hypothesize that the choice of folate indicators may be a contributing factor. In clinical practice, both red blood cell (RBC) folate and serum folate are commonly used to assess folate levels. Although both indicators measure the same substance, their correlation ranges between 0.5 and 0.6, likely due to differences in their forms and detection methods (Youssry and Kamel, 2019; Maruvada et al., 2020). Serum folate reflects the circulating folate levels in the body but is susceptible to factors such as recent dietary intake. In contrast, RBC folate represents the body’s folate stores and is more stable as it is less influenced by short-term dietary changes (Li et al., 2018; Chen et al., 2021). Therefore, we conducted a retrospective study using data from the 2017–2020 National Health and Nutrition Examination Survey (NHANES) to explore the relationship between serum folate, RBC folate, and the prevalence of MASLD.

## 2 Materials and methods

### 2.1 Participant selection

**FIGURE 1:**
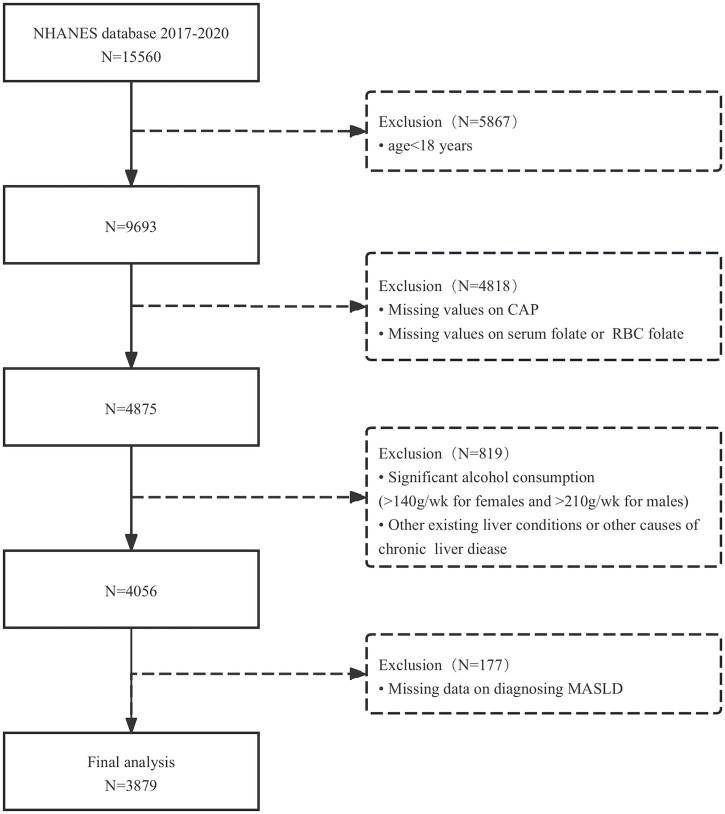
Flowchart of participant selection.

The study utilized data from the 2017–2020 NHANES. NHANES is a biennial research program designed to assess the health and nutritional status of adults and children in the United States. The survey employs a stratified multistage probability sampling design to select non-institutionalized participants. Data categories primarily included demographic characteristics, dietary intake, physical examinations, laboratory tests, and questionnaire responses. From the NHANES database (2017–2020), we initially screened 15,560 participants who underwent controlled attenuation parameter (CAP) assessments for hepatic steatosis and blood folate testing. Exclusion criteria were as follows: 1. age <18 years; 2. refusal or inability to complete CAP examination; 3. missing values for RBC folate or serum folate; 4. significant alcohol consumption (>210 g/week for men or >140 g/week for women); 5. pre-existing liver conditions (e.g., hepatitis B or C infection); and 6. incomplete data for MASLD diagnosis. After applying these criteria, 3,879 participants were included in the final analysis (Figure 1).

### 2.2 Measurement of MASLD

Hepatic steatosis was evaluated using liver ultrasound transient elastography (FibroScan® model 502 V2 Touch) with medium (M) or extra-large (XL) probes. The CAP was used to diagnose SLD, with a detectable range of 100–400 dB/m. Participants were classified as having SLD if their CAP value was ≥ 285 dB/m (Taesuwan et al., 2025). MASLD diagnosis required the presence of SLD (CAP ≥285 dB/m) combined with at least one of the following cardiometabolic risk factors defined by the Delphi Consensus Statement: 1. body mass index (BMI) > 25 kg/m^2^ (or >23 kg/m^2^ for Asian individuals), waist circumference >94 cm (men) or >80 cm (women), or ethnicity-adjusted thresholds; 2. fasting glucose ≥ 5.6 mmol/L, 2-h postprandial glucose ≥ 7.8 mmol/L, HbA1c ≥ 5.7%, or diagnosed/treated type 2 diabetes; 3. blood pressure ≥ 130/85 mmHg or antihypertensive medication use; 4. triglycerides ≥ 1.70 mmol/L or lipid-lowering treatment; and 5. high-density lipoprotein (HDL) cholesterol ≤ 1.0 mmol/L (men) or ≤ 1.3 mmol/L (women) or lipid-lowering therapy.

### 2.3 Alcohol consumption

Alcohol intake was assessed via the NHANES “Alcohol Use” questionnaire, which included items on lifetime alcohol consumption (“ALQ111”), frequency of alcohol use in the past 12 months (“ALQ121”), and average daily alcohol intake (“ALQ130”). One standard drink was defined as 14 g of alcohol (equivalent to 12 oz beer, 5 oz wine, or 1.5 oz liquor). Participants exceeding sex-specific thresholds (men >210 g/week; women >140 g/week) were classified as having significant alcohol consumption. Although MASLD encompasses metabolic and alcohol-associated steatohepatopathy (MetALD), individuals with alcohol intake between 140 and 350 g/week (women) or 210 and 420 g/week (men) were excluded due to the heterogeneity of alcohol-induced liver damage.

### 2.4 Other covariates and definitions

Demographic variables (age, gender, race, and income-to-poverty ratio) and lifestyle factors (smoking status and physical activity) were collected via computer-assisted personal interviews. Dietary data (energy intake and folate intake) and anthropometric measurements (weight, height, waist circumference, and blood pressure) were obtained during mobile examination center (MEC) visits. Dietary energy intake (DEI) and dietary folate intake (DFI) were calculated as the average of two 24-h dietary recalls. Laboratory data included total cholesterol (TC), total glyceride (TG), HDL cholesterol, alanine aminotransferase (ALT), aspartate aminotransferase (AST), uric acid (UA), C-reactive protein (CRP), serum folate, and RBC folate. In particular, population folate status was assessed using a combination of two analytical methods: whole-blood folate was measured using a microbiologic assay, while serum folate forms were measured via isotope-dilution high-performance liquid chromatography coupled to tandem mass spectrometry (LC-MS/MS); RBC folate was then measured using data from both assays (Zhang et al., 2021). Hypertension was defined as systolic blood pressure ≥ 140 mmHg or diastolic blood pressure ≥ 90 mmHg or a history of hypertension. Diabetes was defined as an FPG ≥ 7.0 mmol/L or HbA1c ≥ 6.5% or ever having a history of diabetes.

### 2.5 Statistical analysis

Continuous variables were expressed as median (interquartile range, IQR), and categorical variables were expressed as frequencies (percentages). Group differences (MASLD vs non-MASLD) were analyzed using Kruskal–Wallis tests (continuous) and chi-square tests (categorical). Multivariable logistic regression models were constructed to assess associations between folate levels (serum/RBC) and MASLD, including a crude model (no covariates were adjusted), Model I (adjusted for age, gender, race, and income), Model II (adjusted for covariates in Model I + BMI, physical activity, smoking, hypertension, diabetes, DEI, and DFI), and Model III (adjusted for covariates in Model II + TC, ALT, AST, UA, and CRP). Generalized additive models (GAMs) were used to explore nonlinear relationships. Subgroup analyses stratified by age, gender, and BMI were conducted to evaluate effect modification. All analyses were performed using SPSS 27.0 and R 4.3.2, with statistical significance set at *P* < 0.05 (two-tailed).

## 3 Results

### 3.1 Characteristics of participants

A total of 3,879 participants were included in the final analysis (1,535 male participants and 2,344 female participants), with an overall MASLD prevalence of 34.1%. The median age was 45 years (IQR: 31–61). As shown in Table 1, participants with MASLD were significantly older (median 51 vs. 41 years, *P* < 0.001) and exhibited higher BMI (33.4 vs. 26.4 kg/m^2^, *P* < 0.001) and waist circumference (111.0 vs. 91.9 cm, *P* <0.001) than non-MASLD individuals. Additionally, the MASLD group had a higher prevalence of diabetes (29.4% vs. 8.6%, *P* < 0.001), hypertension (53.2% vs. 31.5%, *P* <0.001), and smoking (41.0% vs. 34.1%, *P* < 0.001). Laboratory findings revealed elevated levels of total cholesterol, triglycerides, ALT, AST, uric acid, and C-reactive protein (*P* < 0.001 for all), alongside reduced HDL cholesterol (*P* < 0.001). Notably, RBC folate levels were significantly higher in the MASLD group (median 494 vs. 446 ng/mL, *P* < 0.001), whereas no significant differences were observed in serum folate, DEI, or DFI (*P* > 0.05).

**TABLE 1:** Characteristics of the participants studied.

Variable	Overall	MASLD		P-value
		No	Yes	
N (%)	3879 (100)	2558 (65.9)	1321 (34.1)	
Age (years)				<0.001
Median (IQR)	45.0 (31.0–61.0)	41.0 (28.0–59.0)	51.0 (38.0–63.0)	
Gender (%)				<0.001
Male	1535 (39.6)	918 (35.9)	617 (46.7)	
Female	2344 (60.4)	1640 (64.1)	704 (53.3)	
Race (%)				<0.001
Mexican American	477 (12.3)	250 (9.8)	227 (17.2)	
Other Hispanic	393 (10.1)	269 (10.5)	124 (9.4)	
Non-Hispanic White	1370 (35.3)	866 (33.9)	504 (38.2)	
Non-Hispanic Black	987 (25.4)	713 (27.9)	274 (20.7)	
Other race	652 (16.8)	460 (18.0)	192 (14.5)	
Income (PIR)				0.293
<1.3	997 (29.2)	668 (29.8)	329 (28.0)	
1.3–3.5	1302 (38.1)	834 (37.2)	468 (39.8)	
>3.5	1120 (32.8)	742 (33.1)	378 (32.2)	
Physical activity				0.004
Inactivate	1040 (26.8)	649 (25.4)	391 (29.6)	
Moderate	1347 (34.7)	884 (34.6)	463 (35.0)	
Vigorous	1492 (38.5)	1025 (40.1)	467 (35.4)	
Smoking (%)				<0.001
Yes	1413 (36.4)	871 (34.1)	542 (41.0)	
No	2466 (63.6)	1687 (65.9)	779 (59.0)	
Hypertension (%)				<0.001
Yes	1510 (38.9)	807 (31.5)	703 (53.2)	
No	2369 (61.1)	1751 (68.5)	618 (46.8)	
Diabetes (%)				<0.001
Yes	608 (15.7)	220 (8.6)	388 (29.4)	
No	3271 (84.3)	2338 (91.4)	933 (70.6)	
Dietary				
DEI (kcal)	1920.0 (1404.0–2540.0)	1893.0 (1375.0–2517.0)	1968.0 (1469.0–2626.8)	0.092
DFI (ug)	302.0 (205.0–435.0)	302.0 (203.0–434.0)	302.0 (207.0–436.0)	0.973
Examination and laboratory				
BMI (kg/m^2^)	28.7 (24.4–34.0)	26.4 (23.0–30.7)	33.4 (29.2–38.8)	<0.001
WC (cm)	98.0 (86.5–111.0)	91.9 (81.6–102.5)	111.0 (101.1–122.4)	<0.001
TC (mg/dL)	179 (155.0–206.0)	176.0 (153.0–203.0)	184.0 (159.0–211.0)	<0.001
TG (mg/dL)	107 (74.0–156.0)	92.0 (68.0–130.0)	141.0 (102.0–202.0)	<0.001
HDL (mg/dL)	51 (42.0–61.0)	54.0 (46.0–65.0)	45.0 (39.0–53.0)	<0.001
ALT (U/L)	17.0 (12.0–24.0)	15.0 (11.0–21.0)	21.0 (15.0–31.0)	<0.001
AST (U/L)	18.0 (15.0–23.0)	18.0 (15.0–22.0)	19.0 (16.0–25.0)	<0.001
UA (umol/L)	303.3 (249.8–356.9)	285.5 (237.9–339.0)	333.1 (279.6–392.6)	<0.001
CRP (mg/L)	1.9 (0.8–4.5)	1.4 (0.6–3.5)	3.2 (1.4–6.4)	<0.001
Serum folate (ng/mL)	14.3 (9.9–21.1)	14.5 (10.0–21.1)	13.9 (9.6–20.9)	0.076
RBC folate (ng/mL)	464.0 (365.0–596.0)	446.0 (355.0–574.0)	494.0 (392.0–640.0)	<0.001

Values were presented as the median (IQR) for continuous variables or as a number (percentage) for categorical variables. Abbreviations: MASLD, metabolic dysfunction-associated steatotic liver disease; IQR, interquartile range; PIR, poverty income ratio; DEI, dietary energy intake; DFI, dietary folate intake; BMI, body mass index; WC, waist circumference; TC, total cholesterol; TG, triglyceride; HDL, high-density lipoprotein; ALT, alanine transaminase; AST, aspartate transaminase; UA, uric acid; CPR, C-reactive protein; RBC, red blood cell.

### 3.2 Associations between serum folate and MASLD

**FIGURE 2:**
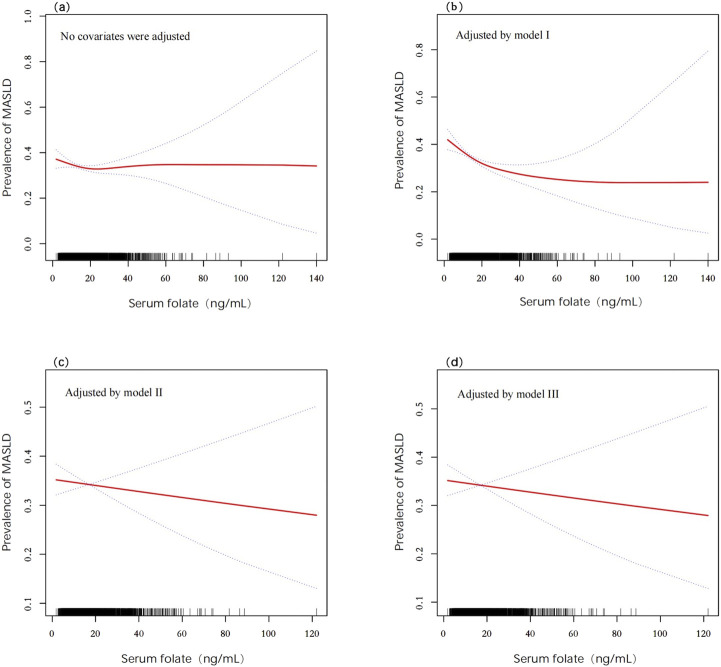
Associations between serum folate and the prevalence of MASLD detected by GAMs after adjusting for different models. **(a)** Crude model (no covariates were adjusted); **(b)** Model I (age, gender, race, and income were adjusted); **(c)** Model II (covariates in Model I with BMI, physical activity, smoking, hypertension, diabetes, DEI, and DFI were adjusted); **(d)** Model III (covariates in Model II with TC, ALT, AST, UA, and CRP were adjusted). Abbreviations: MASLD, metabolic dysfunction-associated steatotic liver disease; DEI, dietary energy intake; DFI, dietary folate intake; BMI, body mass index; TC, total cholesterol; ALT, alanine transaminase; AST, aspartate transaminase; UA, uric acid; CPR, C-reactive protein.

**FIGURE 3:**
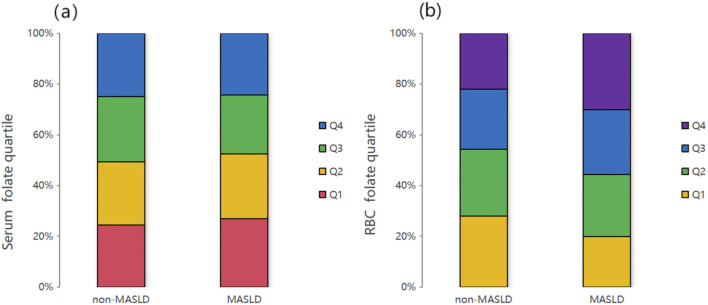
Serum folate **(a)** and RBC folate **(b)** quartiles of all participants from the MASLD and non-MASLD groups. The results showed that the distribution of the RBC folate levels in the MASLD group was relatively higher than that in the non-MASLD group (P < 0.001). However, no obvious difference was observed between the two groups for serum folate quartiles (P = 0.209).

Multivariate logistic regression demonstrated that serum folate was not independently associated with MASLD in the crude model (OR = 0.975 and 95% CI: 0.912–1.044), Model II (OR = 1.013 and 95% CI: 0.923–1.111), or Model III (OR = 1.006 and 95% CI: 0.915–1.106). A transient protective effect was observed in Model I (adjusted for age, gender, race, and income), where each standard unit increase in serum folate reduced the MASLD risk by 13.1% (OR = 0.869, 95% CI: 0.802–0.941, and *P* < 0.001). However, this association dissipated after further adjustments for metabolic and inflammatory markers. Quartile analysis similarly showed no significant trends in serum folate distribution between MASLD and non-MASLD groups (*P* = 0.209) (Figure 3a). GAMs further confirmed nonlinear relationships in crude and Model I analyses, but these patterns were obscured in fully adjusted models (Figures 2a–d).

### 3.3 Associations between RBC folate and MASLD

In contrast, RBC folate exhibited a robust positive correlation with MASLD across all models. As a continuous variable, each standard unit increase in RBC folate elevated MASLD odds by 28.8% in the crude model (OR = 1.288, 95% CI: 1.205–1.376, and *P* < 0.001), 19.2% in Model I (OR = 1.192, 95% CI: 1.106–1.285, and *P* < 0.001), 13.7% in Model II (OR = 1.137, 95% CI: 1.041–1.242, and *P* < 0.01), and 11.1% in Model III (OR = 1.111, 95% CI: 1.015–1.216, and *P* < 0.05). Quartile analysis revealed a dose-dependent relationship: compared to the lowest quartile (Q1), the highest RBC folate quartile (Q4) was associated with a 44.3% increased risk of MASLD in the fully adjusted model (OR = 1.443, 95% CI: 1.113–1.872, and *P* < 0.001) (Table 2). This trend was visually supported by the significantly higher RBC folate distribution in the MASLD group (*P* < 0.001) (Figure 3b).

**TABLE 2:** Relationships between serum/RBC folate and MASLD in different models.

Serum folate	Crude model **(**OR, 95% CI)	Model I **(**OR, 95% CI)	Model II (OR, 95% CI)	Model III (OR, 95%CI)
Per 1 unit increase	0.975 (0.912–1.044)	0.869 (0.802–0.941)***	1.013 (0.923–1.111)	1.006 (0.915–1.106)
Quartile (ng/mL)				
Q1 (<9.9)	Ref	Ref	Ref	Ref
Q2 (9.9–14.3)	0.919 (0.763–1.106)	0.960 (0.784–1.175)	1.311 (1.027–1.674)*	1.183 (0.919–1.523)
Q3 (14.4–21.1)	0.819 (0.679–0.988)	0.734 (0.597–0.902)**	1.060 (0.823–1.365)	0.970 (0.747–1.260)
Q4 (>21.1)	0.882 (0.731–1.063)	0.699 (0.566–0.863)***	1.203 (0.930–1.556)	1.139 (0.874–1.484)
P for trend	0.209	<0.001	0.123	0.333
RBC folate				
Per 1 unit increase	1.288 (1.205–1.376)***	1.192 (1.106–1.285)***	1.137(1.041–1.242)**	1.111 (1.015–1.216)*
Quartile (ng/mL)				
Q1 (<365)	Ref	Ref	Ref	Ref
Q2 (365–464)	1.315 (1.083–1.597)**	1.199 (0.970–1.481)	1.219 (0.946–1.570)	1.080 (0.831–1.403)
Q3 (465–596)	1.521 (1.252–1.848)***	1.382 (1.117–1.709)**	1.331 (1.031–1.718)*	1.179 (0.907–1.533)
Q4 (>596)	1.937 (1.600–2.346)***	1.587 (1.283–1.962)***	1.587 (1.232–2.045)***	1.443 (1.113–1.872)**
P for trend	<0.001	<0.001	0.004	0.032

Logistic regression was used to detect the odds ratio (95% CI); ***P < 0.001, **P < 0.01, and *P < 0.05; Crude model: other co-variants are not adjusted; Model Ⅰ: crude model +age + gender + race + income; Model Ⅱ: model Ⅰ + BMI + physical activity + smoking + hypertension + diabetes + DEI + DFI; Model Ⅲ: Model Ⅱ + TC + ALT + AST + UA + CRP., Abbreviations: MASLD, metabolic dysfunction-associated steatotic liver disease; RBC, red blood cell; BMI, body mass index; DEI, dietary energy intake; DFI, dietary folate intake; TC, total cholesterol; ALT, alanine transaminase; AST, aspartate transaminase; UA, uric acid; CPR, C-reactive protein. All continuous variables (age, BMI, TC, ALT, AST, UA, CRP, DEI, DFI, serum folate, and RBC folate) were processed with standardized regression coefficients to eliminate the impact of different magnitudes.

### 3.4 Subgroup analysis

**FIGURE 4:**
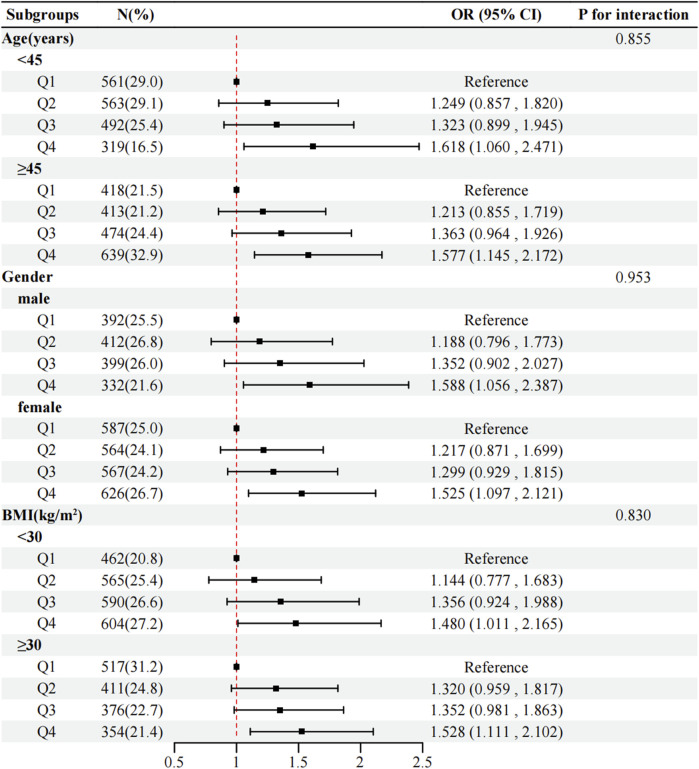
Subgroup regression to assess the relationship between RBC folate and MASLD. Analyses above were adjusted for age, gender, race, income, BMI, smoking, diabetes, hypertension, physical activity, DEI, and DFI. In each case, the model is not adjusted for the stratification variable. Abbreviations: RBC, red blood cell; MASLD, metabolic dysfunction-associated steatotic liver disease; BMI, body mass index; DEI, dietary energy intake; DFI, dietary folate intake.

Subgroup analyses stratified by age, gender, and BMI demonstrated no significant interactions (*P* for interaction > 0.05 for all), indicating that the positive association between RBC folate and MASLD remained consistent across demographic and metabolic subgroups (Figure 4).

## 4 Discussion

This cross-sectional study analyzed NHANES data from 2017 to 2020 to explore the association between two forms of folate (serum folate and RBC folate) and MASLD. Our findings revealed that serum folate demonstrated a negative correlation with MASLD prevalence only in a minimally adjusted model (Model I), but this association lost significance in fully adjusted or crude models. In contrast, RBC folate levels exhibited a consistent positive association with MASLD across all models. In particular, when analyzed as a continuous variable, RBC folate showed a linear relationship with the MASLD risk, where higher concentrations correlated with increased odds of MASLD. Similarly, quartile analyses indicated that higher RBC folate quartiles were associated with elevated MASLD prevalence, even after adjusting for confounders. Subgroup analyses further confirmed that age, gender, and BMI did not modify the relationship between RBC folate and MASLD.

Clinical findings regarding folate and MASLD or NAFLD remain controversial. A study on adult participants in China showed that low serum folate levels have been identified as an independent risk factor for NAFLD, and the incorporation of serum folate levels into the existing NAFLD prediction scores has led to a notable enhancement in the accuracy of NAFLD prediction (Yuan et al., 2022). Guo et al. found that higher serum folate levels were associated with a lower risk of NAFLD through a cross-sectional study of 5,714 American adult participants (Leow et al., 2023). A meta-analysis of homocysteine, folate, and NAFLD by Shuai et al. also found that serum folate was negatively associated with the risk of developing NAFLD (Konyn et al., 2023). On the contrary, in a study utilizing data from NHANES 1999–2004, Li et al. investigated the correlation between vitamin B12 markers and NAFLD. Their findings indicated that RBC folate exerted a significant independent influence on NAFLD, while serum folate demonstrated no association with NAFLD (Gofton et al., 2023). Yalan Chen et al. did not support a causal relationship between serum folate levels and the risk of MASLD (Chen et al., 2024). Our study suggests that MASLD may likely be associated with higher RBC folate levels instead of serum folate, which is consistent with the findings of Li and Chen et al. A negative correlation between serum folate and MASLD was only observed in Model I, which disappeared with the addition of more correction factors. This may be related to the fact that serum folate is more susceptible to confounding factors, whereas RBC folate may be a more stable indicator. It may also be related to different study populations, inclusion and exclusion criteria, and some other potential factors.

Folate is a water-soluble vitamin B9, which is absorbed mainly in the duodenum and proximal jejunum and is stored partly as polyglutamate folate in red blood cells, the liver, and other tissues and partly as monoglutamate folate distributed in plasma, tissue fluids, bile, and urine (Malinowska et al., 2022). There are significant differences in folate metabolism in erythrocytes and serum. Folate in erythrocytes is predominantly in the form of 5-methyltetrahydrofolate and is taken up during erythropoiesis, so folate levels in erythrocytes reflect long-term folate status rather than short-term dietary changes (Anderson et al., 2013). In contrast, serum folate levels are more susceptible to recent dietary intake, especially the use of folate supplements (Stamm et al., 2018). Folate intake is important for the maintenance of health. Folate deficiency during the first 3 months of pregnancy can lead to neural tube defects (NTDs) in the foetus and a chronic deficiency can cause macrocytic anemia. Although folate supplementation through dietary medication can significantly reduce the incidence of NTDs, some studies have shown that excessive intake of folate can also have adverse effects on the body (Maher and Sobczyńska-Malefora, 2021). Bailey R et al. showed that more than 1 mg of folate per day significantly increased serum levels of unmetabolized folate (UMFA) and that increased UMFA inhibited hepatic dihydrofolate reductase activity, further reducing folate metabolism and clearance (Liu et al., 2021). In another study, researchers found that folic acid supplement intake was associated with an increase in red blood cell folate concentrations, especially in individuals with a specific genotype (Anderson et al., 2013). This suggests that increased folate intake may lead to significant changes in erythrocyte folate levels, especially in certain genetic backgrounds. Huang et al. found that individuals with high levels of folate were associated with an increased risk of benign prostatic hyperplasia (Malinowska et al., 2022). Daniel L et al. showed that excessive folate intake can disrupt the internal balance of cholesterol in the liver (Paternostro and Trauner, 2022). Some other studies have shown that higher RBC folate concentrations are strongly associated with the development of gestational diabetes and may also lead to an increased risk of obesity and type 2 diabetes (Li et al., 2022; Zhou et al., 2022). A review concluded that excessive intake of folic acid increases the risk of cancer, disrupts DNA methylation—leading to abnormalities in red blood cell maturation, embryonic development, and neurodevelopment—and increases the risk of gestational diabetes mellitus (Fardous and Heydari, 2023). These abovementioned findings suggest that we should be careful to take the right amount of folic acid supplementation to avoid potential health risks.

The exact mechanism of MASLD development is unclear and is mainly related to various factors such as oxidative stress, gut microbiology, lipid metabolism, genetic susceptibility, insulin resistance, and some nutritional and lifestyle factors (Byrne and Targher, 2015; Rives et al., 2020). There have also been several studies on the relationship between folate and the occurrence of MASLD. A study from Guo et al. found that a folic acid overdose in mice resulted in a disorder of hepatic lipid metabolism, characterized by increased fat synthesis and decreased lipolysis. This metabolic disturbance may be due to the inhibition of the one-carbon metabolic pathway by folate and altered expression of related genes (Guo et al., 2024). Another study also noted that excessive folic acid intake may affect hepatic lipid metabolism by altering the composition and function of the gut microbiota, which, in turn, affects hepatic lipid metabolism. This gut–liver axis interaction may play an important role in the onset and development of MASLD (Yao et al., 2022). Oxidative stress occurs in the livers of MASLD patients, and excess folate can lead to an increase in intracellular reactive oxygen species, which promotes MASLD (Sanyal et al., 2023; Tincopa and Loomba, 2023). UMFA can accumulate in the body when folate intake is too high, and some studies have found that UMFA is significantly associated with a higher prevalence of MASLD and that it can increase the concentration of relevant pro-inflammatory factors and reduce the cytotoxicity of natural killer cells, which may lead to the development of MASLD (Fardous and Heydari, 2023; Chen and Huang, 2024; Kim et al., 2024). However, Shao et al. indicated that a higher intake of folate did not show any significant correlation with NAFLD or related liver fibrosis (Teng et al., 2023). In the present study, we found that the level of DFI was not associated with the occurrence of MASLD, which is consistent with the findings of Shao et al. In addition, folate levels may be affected by other nutrients and metabolites. For example, vitamin B12 and homocysteine levels are strongly associated with folate metabolism. It has been shown that B vitamin imbalance due to high folate and relative vitamin B12 deficiency can lead to functional folate deficiency, which triggers insulin resistance and gestational diabetes. In addition, insulin resistance or diabetes is one of the major risk factors for MASLD (Chen et al., 2023; En Li Cho et al., 2023). Some studies have shown that serum folate deficiency can lead to hyperhomocysteinemia, which increases the risk of hypertension and cardiovascular disease, while high homocysteine levels are also an independent risk factor for liver fibrosis and cirrhosis (Yang et al., 2024). Considering these factors together can help us better understand and manage folate levels in red blood cells and serum.

The main strengths of this study are that it used the results of the NHANES survey, which has a large sample size, and that it assessed the correlation between two indicators of folate (serum and erythrocyte) and the prevalence of MASLD. Additionally, this study used RBC folate as an indicator that better reflects long-term folate levels *in vivo* than serum folate, and rationalization of covariates and stratified analyses made the conclusions more reliable. However, there are still some limitations that cannot be ignored. First, the nature of this study was cross-sectional, and we could not determine a causal relationship between RBC folate and MASLD. Second, we used CAP results obtained by ultrasound elastography as a criterion for the diagnosis of MASLD rather than the gold standard liver biopsy, and its accuracy and cut-off value are somewhat controversial. Third, although we included as many covariates as possible to exclude bias in confounding factors, other potential factors may still cause bias. Accordingly, further prospective studies are required to elucidate the detailed relationship between RBC folate and MASLD.

## 5 Conclusion

This study demonstrates a significant positive association between RBC folate levels and the prevalence of MASLD, with a clear dose-response trend observed across increasing quartiles of RBC folate. In contrast, serum folate exhibited inconsistent associations, showing a transient negative correlation only in partially adjusted models. These findings suggest that RBC folate, reflecting long-term folate status, may serve as a more reliable biomarker than serum folate in studying MASLD risk. Future prospective studies and mechanistic research are warranted to validate these associations and elucidate the underlying biological pathways linking elevated RBC folate to MASLD development.

## Data Availability

The datasets presented in this study can be found in online repositories. The names of the repository/repositories and accession number(s) can be found in the article/supplementary material.
